# Prenatal cigarette smoke exposure sensitizes acetaminophen-induced liver injury by modulating miR-34a-5p in male offspring mice

**DOI:** 10.3389/fcell.2024.1393618

**Published:** 2024-07-30

**Authors:** Daram Yang, Hyuneui Jeong, Min-Seok Kim, Sang-Ik Oh, Kyuhong Lee, Jong-Won Kim, Bumseok Kim

**Affiliations:** ^1^ Biosafety Research Institute and College of Veterinary Medicine, Jeonbuk National University, Iksan, Republic of Korea; ^2^ Inhalation Toxicology Center, Jeonbuk Department of Inhalation Research, Korea Institute of Toxicology, Jeongeup, Jeonbuk, Republic of Korea; ^3^ Center for Pharmacogenetics and Department of Pharmaceutical Sciences, University of Pittsburgh, Pittsburgh, PA, United States

**Keywords:** microRNA, prenatal exposure, cigarette smoke, acute liver injury, acetaminophen

## Abstract

**Introduction:** Cigarette smoke (CS) exacerbates the severity of diseases not only in lungs, but also in systemic organs having no direct contact with smoke. In addition, smoking during pregnancy can have severe health consequences for both the mother and the fetus. Therefore, our aim was to evaluate effects of prenatal exposure to CS on acetaminophen (APAP)-induced acute liver injury (ALI) in offspring.

**Methods:** Female C57BL/6 mice on day 6 of gestation were exposed to mainstream CS (MSCS) at 0, 150, 300, or 600 μg/L for 2 h a day, 5 days a week for 2 weeks using a nose-only exposure system. At four weeks old, male offspring mice were injected intraperitoneally with a single dose of APAP at 300 mg/kg body weight to induce ALI.

**Results:** Maternal MSCS exposure significantly amplified pathological effects associated with ALI as evidenced by elevated serum alanine aminotransferase levels, increased hepatocellular apoptosis, higher oxidative stress, and increased inflammation. Interestingly, maternal MSCS exposure reduced microRNA (miR)-34a-5p expression in livers of offspring. Moreover, treatment with a miR-34a-5p mimic significantly mitigated the severity of APAP-induced hepatotoxicity. Overexpression of miR-34a-5p completely abrogated adverse effects of maternal MSCS exposure in offspring with ALI. Mechanistically, miR-34a-5p significantly decreased expression levels of hepatocyte nuclear factor 4 alpha, leading to down-regulated expression of cytochrome P450 (CYP)1A2 and CYP3A11.

**Discussion:** Prenatal exposure to MSCS can alter the expression of miRNAs, even in the absence of additional MSCS exposure, potentially increasing susceptibility to APAP exposure in male offspring mice.

## Graphical Abstract

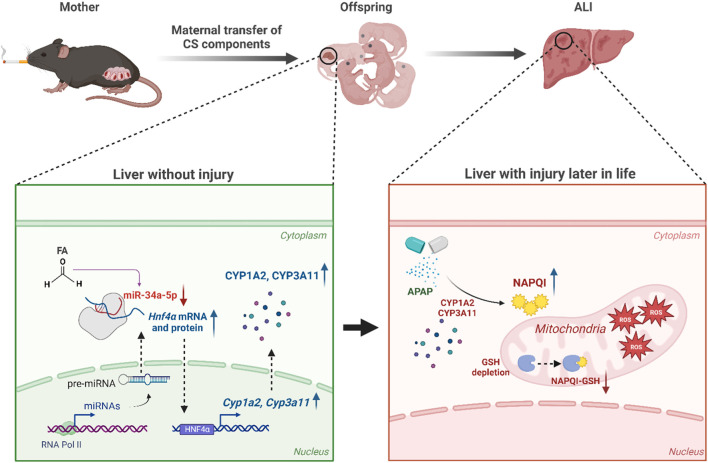

## Introduction

Acetaminophen (N-Acetyl-p-Aminophenol; APAP) is a commonly used analgesic and antipyretic drug. It is generally considered safe at therapeutic doses. However, overdose of APAP can result in severe hepatotoxicity, posing a significant threat to human health (Chun et al., 2009). Therefore, understanding mechanisms underlying APAP-induced hepatotoxicity in mice is crucial because mice are well-established experimental models with significant clinical relevance. Upon ingestion, APAP undergoes biotransformation mediated by cytochrome P450 (CYP) enzymes in the liver, a major organ involved in drug metabolism and detoxification. This process results in formation of two major metabolites: a non-toxic 3-hydroxy-APAP and a highly reactive metabolite N-acetyl-p-benzoquinone-imine (NAPQI) (Hazai et al., 2002). NAPQI is known to cause hepatocellular damages and can be detoxified through conjugation with hepatic glutathione (GSH) (Dahlin et al., 1984), a potent antioxidant that plays an important role in improving cellular stress caused by reactive oxygen species (ROS). However, in case of APAP overdose, the available pool of GSH becomes limited in the liver, resulting in binding of unconjugated NAPQI to cellular proteins (Mitchell et al., 1973), leading to mitochondrial dysfunction and hepatocellular necrosis (Larsen and Wendon, 2014).

MicroRNAs (miRNAs) are short, endogenously initiated non-coding RNAs. They exert their regulatory effects by binding to target mRNA sequences, leading to mRNA degradation or post-transcriptional translation inhibition (Cai et al., 2009). A single miRNA can control the expression of multiple potential target mRNAs, and individual mRNAs can be intricately regulated by multiple miRNAs (Kuhn et al., 2008). Accumulating evidence has revealed the crucial role of miRNAs as regulators in various cellular processes, including cell proliferation, growth, differentiation, and tumorigenesis (Harfe, 2005; Cai et al., 2009; Ganju et al., 2017). Therefore, miRNAs play a crucial role in the pathogenesis of acute or chronic diseases in various organs, including the liver. Recent evidence has demonstrated that miRNAs derived from multiple cell types in the liver can contribute to the development and progression of liver diseases such as nonalcoholic steatohepatitis, liver fibrosis, and hepatocellular carcinoma (HCC) (Doghish et al., 2023). Moreover, specific miRNAs have been closely associated with APAP-induced acute liver injury (ALI). For instance, miR-122 and miR-192, which are highly expressed in hepatocytes, have been identified as potential biomarkers for liver injury caused by APAP overdose (Starkey Lewis et al., 2011). Notably, miR-122 can exert a protective function in APAP-induced hepatotoxicity by modulating CYP1A2 and CYP2E1 (Chowdhary et al., 2017). Additionally, depletion of miR-194 or miR-21 can promote liver regeneration and reduces APAP-induced hepatotoxic injury (Chang et al., 2022; Huffman et al., 2023). Considering these studies, regulating endogenous miRNAs in the context of ALI is suggested as a crucial therapeutic approach to alleviate liver injury.

Cigarette smoke (CS) is a known cause of premature death, with its harmful effects being emphasized annually. CS emitted from burning cigarettes has been recognized as a significant contributor to exacerbation of diseases, not only within the respiratory system but also in systemic organs that lack direct exposure to CS itself (Petrik et al., 1995). It has been reported that smoking can aggravate the development of hyperplasia in a rat carotid artery intimal injury model and accelerate the progression of kidney failure in diabetic patients and those with alcoholic chronic pancreatitis (Maisonneuve et al., 2005; Yacoub et al., 2010). Moreover, numerous studies have linked CS to detrimental effects on liver pathologies including an increased incidence of HCC in patients with chronic liver disease (El-Zayadi, 2006). A significant positive correlation of 4-aminobiphenyl, a hepatic carcinogen in CS, with HCC risk has been shown in humans (Wang et al., 1998; El-Zayadi, 2006). Furthermore, concern regarding CS during pregnancy has increased due to adverse effects of CS on both the mother and the offspring. Toxic components of CS, such as nicotine, carbon monoxide, and 4-aminobiphenyl, can affect placental vasculature and cross the placenta to the fetus (Harris, 1996; Pastrakuljic et al., 1998). Interestingly, maternal smoking has been found to disproportionately affect male fetal growth compared to female fetal growth (Zaren et al., 2000).

Although exposure to CS has been shown to impact the onset and progression of diseases by regulating the expression of miRNAs in various organs (Izzotti et al., 2018), the association between maternal CS exposure and miRNA expression in the liver of offspring has not been fully investigated and our understanding of how altered miRNA expression influences liver disease in offspring mice is still incomplete. This study aimed to assess the impact of maternal CS exposure on APAP-induced ALI in male offspring mice. In addition, we tried to evaluate the most significantly regulated miRNA in the liver of offspring by maternal CS exposure. We also aimed to determine whether regulating miRNA could modulate APAP-induced ALI of male offspring mice.

## Materials and methods

### Animals

Animal maintenance and experimental procedures were conducted in accordance with the guidelines set by the Animal Care and Ethics Committees of the Korea Institute of Toxicology (KIT; Dae-Jeon, Republic of Korea) and Jeonbuk National University. The animal facility at KIT is fully accredited by the National Association of Laboratory Animal Care. Pregnant female C57BL/6 mice on the third day of gestation were purchased from Central Lab Animal Inc. (Seoul, Korea). All mice were fed a sterile standard diet. They had unrestricted access to water. They were housed under controlled conditions with a temperature of 24°C ± 2°C and a relative humidity of 50% ± 5%, ensuring a pathogen-free environment. A 12-h/12-h light/dark cycle was consistently maintained.

### Experimental protocol

Reference cigarette 3R4F was obtained from Kentucky Tobacco Research and Development Center, University of Kentucky. All cigarettes were conditioned for at least 48 h prior to use at 22°C ± 1°C with a relative humidity of 60% ± 2%, in accordance with International Organization of Standardization (ISO) standard 3,402 (ISO, 1999). CS was generated using a 30-port smoking machine (JB 2080, CH Technologies Inc., Westwood, NJ, United States) following the ISO standard 3,308 smoking protocol (ISO, 2012).

On day 6 after pregnancy, pregnant dams were exposed to mainstream cigarette smoke (MSCS; 150, 300, or 600 μg/L) or filtered clean air for 2 h a day, 5 days a week for 2 weeks using a nose-only exposure system (Nose-only Inhalation Chamber NITC-36, HCT, Korea). They were divided into one control group and three experimental groups: MSCS non-exposed mice (control group, Cont), mice exposed to a low dose of MSCS (CS150), mice exposed to a medium dose of MSCS (CS300), and mice exposed to a high dose of MSCS (CS600). Male offspring mice were studied at postnatal week 4.

To induce acute liver injury, 4-week-old male offspring mice from each exposure group received an intraperitoneal (i.p.) injection of APAP (Sigma-Aldrich, MO, United States) at a dose of either 300 or 500 mg/kg body weight or an equal volume of phosphate-buffered saline (PBS) after 16 h of fasting. The number of mice used per group was as follows for the CS0, CS150, CS300, and CS600 groups in that order: 6, 4, 6, and 6 mice in the PBS group; 7, 6, 7, and 7 mice in the APAP group.

To demonstrate effects of miR-34a-5p on ALI, 4-week-old male offspring mice were administered a single intravenous (i.v.) injection with a Custom Designed SAMIRNA (Bioneer, Daejeon, Korea); mmu-miR-34a-5p (Accession number: MIMAT0000542) mimic SAMIRNA (1 or 10 nmol/mouse) or a negative control SAMIRNA (miR-mock) at 24 h before APAP (300 mg/kg) injection. In experiments using low concentrations of SAMIRNA (1 nmol/mouse), 9 to 11 mice were used per group, and in experiments using high concentrations (10 nmol/mouse), 5 mice were used per group. Sequence information was sourced from miRBase (https://www.mirbase.org/) and a paper by Wang et al. (2016): miR-34a-5p sense, UGG​CAG​UGU​CUU​AGC​UGG​UUG​U; miR-34a-5p antisense, AAU​CAG​CAA​GUA​UAC​UGC​CCU; negative control (NC) sense, UUC​UCC​GAA​CGU​GUC​ACG​U = tt; and NC antisense; ACG​UGA​CAC​GUU​CGG​AGA​A = tt. Mice were anesthetized with a combination of tiletamine hydrochloride and zolazepam hydrochloride (Zoletil 50; Virbac, Carros, France) at a dose of 0.2 mL/kg body weight at 24 h after APAP injection. Blood was collected from the heart for analysis. Liver tissues were then harvested from mice euthanized by cervical dislocation and stored in a RNAlater solution (Sigma-Aldrich) for subsequent analysis. Brief experimental procedures are shown in the Supplementary Figure S1.

### Microarray

To check RNA quality, RNA purity and integrity were evaluated based on OD 260/280 ratio and analyzed using an Agilent 2,100 Bioanalyzer (Agilent Technologies, Palo Alto, United States). Affymetrix Genechip miRNA array process was conducted in accordance with the manufacturer’s protocol. For this, 500 ng RNA samples were labeled using a FlashTag™ Biotin RNA Labeling Kit (Genisphere, Hatfield, PA, United States). Labeled RNA was quantified, fractionated, and hybridized to miRNA microarray according to standard procedures provided by the manufacture. After labeling, RNA was heated to 99°C for 5 min followed by 45°C for 5 min. RNA-array hybridization was performed with agitation at 60 rotations per minute for 16–18 h at 48°C on an Affymetrix® 450 Fluidics Station. Subsequently, chips were washed and stained using a Genechip Fluidics Station 450 (Affymetrix, Santa Clara, CA, United States). These chips were then scanned with an Affymetrix GCS 3000 7G scanner (Affymetrix). Signal values were computed using the Affymetrix^®^ GeneChip™ Command Console software (listed in the Supplementary Table S1).

### Biochemical analysis

Serum was obtained by centrifugation at 1,000 g for 15 min at 4°C. Alanine aminotransferase (ALT) level in the serum was measured using an AM101-K spectrophotometric assay kit (ASAN Pharmaceutical, Seoul, Korea). An EMax spectrophotometer (Molecular Devices, Sunnyvale, CA, United States) was used to determine the optical density at a wavelength of 490 nm.

### Measurement of hepatic malondialdehyde (MDA) and GSH

Hepatic content of MDA, an oxidative stress marker, was determined using a commercially available OxiSelect TBARS Assay Kit (Cell Biolabs Inc., San Diego, CA, United States). Hepatic GSH contents were measured using a GSH quantification kit (Dojindo, Kumamoto, Japan) according to the manufacturer’s instructions.

### Histopathologic examination

Liver tissues were preserved in 10% phosphate-buffered formalin for fixation and subsequently embedded in paraffin. Paraffin blocks were cut into 4 µm thick sections using a microtome (HM-340E, Thermo Fisher Scientific Inc.). These sections were stained with hematoxylin and eosin (H&E) according to standard techniques. To detect hepatocellular apoptosis, the Terminal deoxynucleotidyl transferase-mediated dUTP nick-end labeling (TUNEL) assay was performed using an ApopTaq Peroxidase *in situ* apoptosis detection kit (EMD Millipore, CA, United States) in accordance with the manufacturer’s instructions. TUNEL-positive area per total area was quantified as a percentage. Stained liver sections were evaluated using a light microscope (BX53F, Olympus Corp., Tokyo, Japan) and digital image software program (cellSens Standard, Olympus Corp.).

### Immunohistochemistry (IHC)

Liver tissues were preserved in 10% phosphate-buffered formalin for fixation and then embedded in paraffin. Liver sections were cut and placed onto glass slides. Before staining, slides were deparaffinized, rehydrated, and submerged in an antigen retrieval solution (Dako, Jena, Germany) for 30 min at 100°C. Non-specific binding was blocked with a 3% peroxidase solution, followed by blocking with Super Block (ScyTek Laboratories, Inc., Logan, UT, United States). These sections were then incubated with an anti-myeloperoxidase (MPO) antibody (Abcam, Cambridge, United Kingdom) or an anti-4 hydroxynonenal (4-HNE) antibody (Abcam) overnight at 4°C. Primary antibodies were diluted 1:30 (MPO) and 1:200 (4-HNE) in an antibody diluent. Subsequently, these sections were further incubated with a horse anti-mouse/rabbit IgG antibody (ImmPRESS Universal Polymer Kit Peroxidase; Vector Laboratories, Burlingame, CA, United States). Immune complexes were visualized using a DAB Substrate Kit (Vector Laboratories) according to the manufacturer’s instructions. Finally, tissues were counterstained with 50% hematoxylin and mounted. Images were analyzed under a light microscope (BX53F, Olympus Corp.) with a digital imaging software program (analySIS TS, Olympus Corp.).

### Enzyme-linked immunosorbent assay (ELISA)

To confirm hepatic levels of pro-inflammatory cytokines, such as tumor necrosis factor alpha (TNFα) and interleukin 1 beta (IL1β), protein samples were prepared and measured using ELISA kits (Invitrogen, CA, United States) in accordance with the manufacturer’s protocol.

### Quantitative real time polymerase chain reaction (qRT-PCR)

Total RNAs were isolated from liver tissues using a RiboEx (GeneAll Biotechnology Co. Ltd., Seoul, Korea) and a Hybrid-R RNA purification kit (GeneAll Biotechnology Co. Ltd.). These RNAs were treated with DNase I with an RNase inhibitor (TOYOBO, Osaka, Japan). Reverse transcription was performed using a ReverTra Ace qPCR RT Master Mix (TOYOBO) in accordance with the manufacturer’s instructions to obtain cDNAs. These cDNAs were used for qRT-PCR on a CFX96™ Real-Time PCR Detection System (Bio-Rad Laboratories, CA, United States) with a SYBR Green Master Mix (TOYOBO). Glyceraldehyde-3-phosphate dehydrogenase was used as a reference gene to calculate relative mRNA expression levels. PCR primer sequences are detailed in Table 1.

**TABLE 1:** Used primer sequences for quantitative real-time polymerase chain reaction (qRT-PCR) analysis.

Gene	Forward (5′–3′)	Reverse (5′–3′)	Ref Seq
*Tnfα*	AGG​GTC​TGG​GCC​ATA​GAA​CT	CCA​CCA​CGC​TCT​TCT​GTC​TAC	NM_013693
*Il1β*	CTC​GCA​GCA​GCA​CAT​CAA​CA	CCA​CGG​GAA​AGA​CAC​AGG​TA	NM_008361
*Cyp2e1*	AAGCGCTTCGGGCCAG	TAG​CCA​TGC​AGG​ACC​ACG​A	NM_021282
*Cyp3a11*	CGC​CTC​TCC​TTG​CTG​TCA​CA	CTT​TGC​CTT​CTG​CCT​CAA​GT	NM_007818.3
*Cyp1a2*	GGT​CAG​AAA​GCC​GTG​GTT​G	GAC​ATG​GCC​TAA​CGT​GCA​G	NM_009993
*Gapdh*	ACG​GCA​AAT​TCA​ACG​GCA​CAG	AGA​CTC​CAC​GAC​ATA​CTC​AGC​AC	NM_001289726

CYP, Cytochrome P450; *Gapdh*, glyceraldehyde-3-phosphate dehydrogenase; *Il1β*, interleukin 1 beta; *Tnfα*, tumor necrosis factor alpha.

MiRNAs were isolated from liver tissues using a Hybrid-R™ miRNA kit (GeneAll). These isolated miRNAs were reverse transcribed using a TaqMan™ MicroRNA Reverse Transcription Kit (Applied Biosystems, Foster City, CA) and a TaqMan™ MicroRNA Assay (Applied Biosystems; miR-34a-5p and U6 snRNA) according to the manufacturer’s protocol. miRNAs were quantified by qRT-PCR using a TaqMan™ MicroRNA Assay and a TaqMan™ Universal PCR Master Mix (Applied Biosystems). U6 snRNA was used as an internal control for relative quantification of miRNA expression levels.

### Western blot assay

Liver tissues were lysed using a protein extraction reagent (Thermo Fisher Scientific). After centrifugation at 13,000 × g for 15 min at 4°C, protein extracts were obtained. Protein concentrations were measured using a Pierce BCA Protein Assay kit (Thermo Fisher Scientific). Equal amounts of proteins were electrophoresed on a 10% SDS-PAGE gel and then transferred to a polyvinylidene difluoride membrane that had been activated with methanol. Following the transfer, membranes were blocked with Superblock (Thermo Fisher Scientific) for 1 h at room temperature and then incubated overnight at 4°C with the following primary antibodies: phospho-c-Jun N-terminal kinase (p-JNK), JNK (Cell Signaling Technology, MA, United States), hepatocyte nuclear factor 4 alpha (HNF4α) (Abcam), CYP2E1, CYP1A2, CYP3A11, and β-actin (Santa Cruz Biotechnology, CA, United States). Primary antibodies were diluted from 1:500 to 1:1,500 in a blocking solution. After washing with Tris-buffered saline with 0.1% Tween^®^ 20 Detergent (TBS-T) three times, membranes were incubated with horseradish peroxidase-conjugated secondary IgG antibodies (Enzo Life Sciences, NY, United States) diluted to 1:5,000 in a blocking solution for 2 h at room temperature. Following four washes with TBS-T, these membranes were visualized using a Western ECL Kit (LPS solution, Daejeon, Korea) and captured with ImageQuant LAS 500 (GE Healthcare Life Science, PA, United States). Image analysis to determine relative intensity was conducted using ImageQuant TL software version 8.1 (GE Healthcare Life Science).

### Isolation of primary hepatocytes

Primary hepatocytes were isolated from mice, following a previously described protocol (Kim et al., 2018). Mouse livers were digested with collagenase IV (1 mL/min) (Worthington Biochemical Corporation, Lakewood, NJ, United States) via cannulation of the vena cava. Suspended liver cells were centrifuged at 50 g for 3 min. After centrifugation, the pellet containing hepatocytes was re-suspended, filtered, and washed several times with Dulbecco’s Modified Eagle Medium (DMEM; Lonza, Basel, Switzerland) supplemented with 10% fetal bovine serum (FBS; Thermo Fisher Scientific) and antibiotics-antimycotic solution (Thermo Fisher Scientific). These isolated hepatocytes were then plated onto collagen-coated plates (Sigma-Aldrich). After allowing for cell adhesion at 37°C, media were replaced with fresh serum-free DMEM. Cells were then incubated overnight at 37°C in a 5% CO2 incubator, for further experiments.

### Cell cytotoxicity assay and treatment

Primary hepatocytes were seeded into 96-well plates (100 μL per well, 1 × 10^4^ cells/well). These cells were treated with various concentrations of N-Nitrosodimethylamine (DEN; Sigma-Aldrich), formaldehyde (FA; Sigma-Aldrich), and mirVana™ miR-34a-5p mimic (Cat#: 4464066; Ambion, Austin, TX, United States). They were treated with indicated concentration of DEN, FA, and miR-34a-5p with or without 5 mM APAP. Hepatocellular cytotoxicity was evaluated by measuring the amount of lactate dehydrogenase (LDH) leaked into the culture medium using a Cytotoxicity Detection KitPLUS (Sigma-Aldrich) according to the manufacturer’s instruction. The absorbance of each sample was quantified at a wavelength of 490 nm using an EMax spectrophotometer (Molecular Devices).

Primary hepatocytes (5 × 10^5^ cells/well) were plated into 12-well plates and cultured overnight at 37°C in a 5% CO2 incubator with serum-free DMEM. To mimic cigarette exposure *in vitro*, primary hepatocytes were treated with DEN and FA as representative cigarette components. Concentration that reduced the expression of miR-34a-5p without toxicity were selected. miR-34a-5p mimic was used to restore the expression of miR-34a-5p, which was reduced by cigarette components in primary hepatocytes. To evaluate effects of cigarette components on miR-34a-5p expression *in vitro*, FA (300 μM) was used to treat cells for 24 h. After 24 h of treatment, miR mimic NC (miR-mock; Cat#: 4464058; Ambion) or miR-34a-5p mimic in Opti-MEM (GIBCO, Invitrogen, United States) was used to treat cells for 6 h. Media were then replaced with serum-free DMEM. The following day, cells were exposed to 5 mM APAP for 24 h. Supernatants were then collected for future analysis.

### Statistical analysis

All data are presented as mean ± standard deviation (SD). They were compared using one-way analysis of variance (ANOVA) or two-way ANOVA, followed by Tukey’s multiple comparisons tests using GraphPad Prism 7 (GraphPad Software, San Diego, CA, United States). Liver histopathological assessment was performed using the Kruskal–Wallis nonparametric test, followed by Dunn’s multiple comparisons test. Statistically significance was set at *p* < 0.05.

## Results

### Maternal MSCS exposure directly modulates hepatic protein expression of CYPs in male offspring mice

**FIGURE 1:**
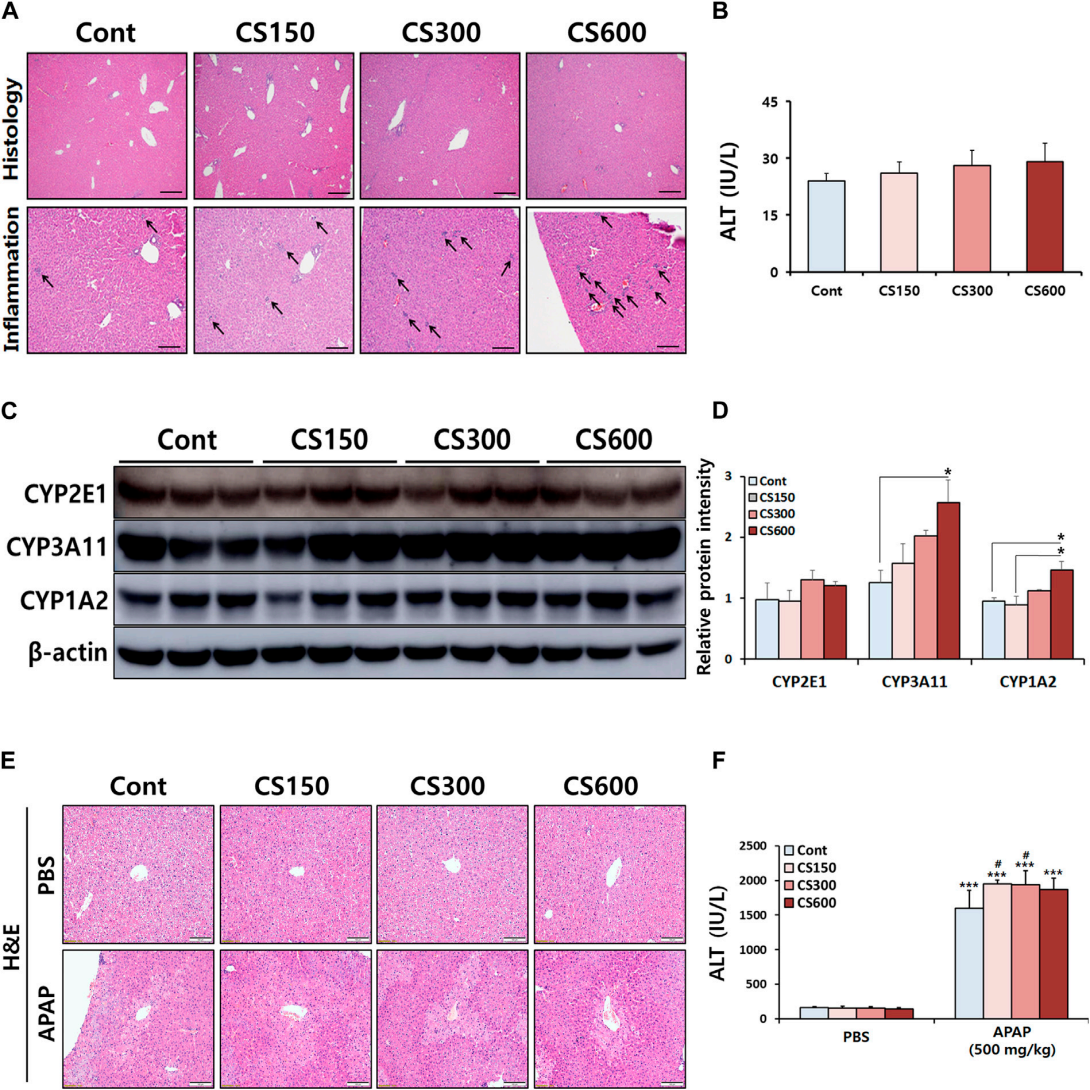
Maternal MSCS exposure directly modulates hepatic protein expression of CYPs in male offspring mice. Pregnant mice were exposed to MSCS (0, 150, 300, or 600 μg/L) for 2 h a day, 5 days a week for 2 weeks from day 6 to day 17 of gestation with a nose-only exposure system. Male offspring mice at 2 weeks old, were euthanized to assess effects of prenatal MSCS exposure. **(A)** Representative histological liver sections stained with H&E (Scale bar: 100 μm). **(B)** Serum ALT levels. **(C)** Hepatic protein levels of CYP enzymes determined by Western blotting, and **(D)** the relative protein intensity was quantified. **(E,F)** Male offspring mice at 4 weeks old were subjected to a single i.p. injection with APAP (500 mg/kg). Representative histological liver sections stained with H&E are shown (Scale bar: 100 μm) and serum ALT levels were measured. Data are presented as mean ± SD. *p < 0.05 in Panel D (one-way ANOVA). ***p < 0.001 *versus* Cont group in the PBS group and #*p* < 0.05 *versus* Cont group in the APAP group in Panel F (two-way ANOVA).

To evaluate effects of maternal MSCS exposure on hepatotoxicity in male offspring, a pre-necropsy examination was conducted 2 weeks after birth. We found no significant difference in body or liver weights of offspring mice with prenatal exposure to MSCS (data not shown). Although the assessment of liver injury, as measured by serum ALT levels, did not exhibit any significant differences among experimental groups, histological analysis of livers revealed notable occurrence of immune cell infiltration in mice exposed to MSCS during the prenatal period (Figures 1A,B). It is already known that several ingredients in CS can modulate the expression of CYP isoforms in various organs (Hukkanen et al., 2011). This led us to test whether prenatal exposure to CS could affect their expression in livers of male offspring mice. Indeed, Western blotting analysis of CYP isoforms involved in drug metabolism showed significant increases of CYP3A11 and 1A2 protein expressions levels in the CS600 group. However, no significant alteration in protein expression of CYP2E1 was observed (Figures 1C,D). Based on these findings, we hypothesized that prenatal exposure to CS might influence the pathogenesis of hepatotoxin- or drug-induced hepatotoxicity in offspring mice, since CYP enzymes such as CYP3A11 and 1A2 plays a vital role in the metabolism of various hepatotoxins of drugs.

To test this hypothesis, male offspring mice received a single i.p. injection of APAP at a dose of 500 mg/kg body weight. Twelve hours post-APAP administration, these mice were euthanized. Interestingly, prenatal exposure to MSCS exacerbated APAP-induced liver injury in male offspring mice. However, this exacerbation did not follow a dose-dependent pattern (Figures 1E,F). Based on these findings, we decided to reduce the APAP dose to 300 mg/kg body weight. Liver tissues were then harvested at 24 h after APAP administration for further experiment.

### Maternal MSCS exposure accelerates APAP-induced liver injury in male offspring mice

**FIGURE 2:**
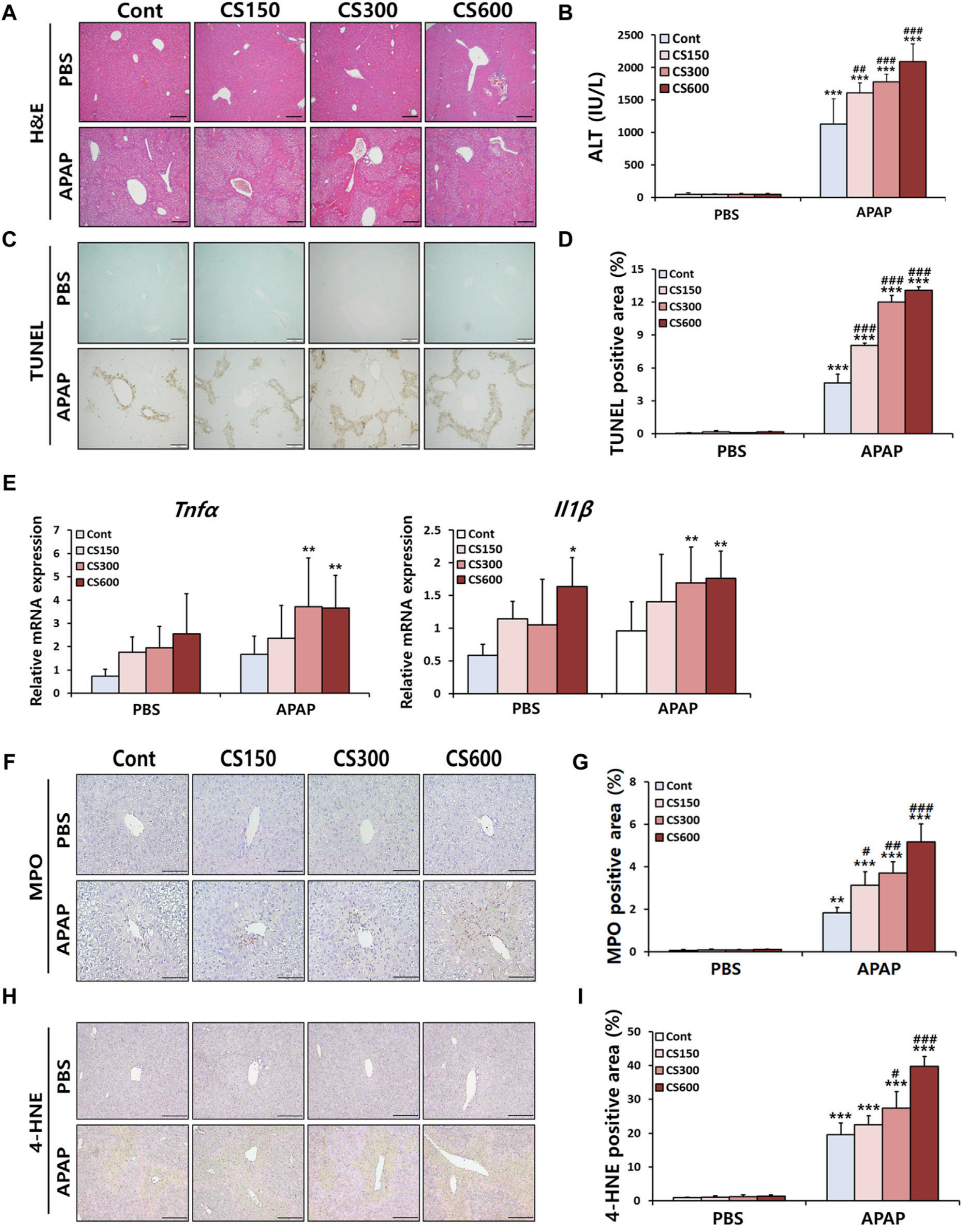
Maternal MSCS exposure accelerates APAP-induced liver injury in male offspring mice. Male offspring mice at 4 weeks old received a single i.p. injection of APAP (300 mg/kg). **(A)** Representative histological liver sections stained with H&E (Scale bar: 100 μm). **(B)** Serum ALT levels. **(C)** Representative histological liver sections stained with TUNEL (Scale bar: 100 μm). **(D)** Positive area graph of TUNEL. **(E)** Hepatic mRNA levels of pro-inflammatory cytokines determined by qRT-PCR. **(F)** Representative images of MPO staining (Scale bar: 100 μm). **(G)** A graph showing percentage of MPO-positive area to total area. **(H)** Representative images of 4-HNE staining (Scale bar: 100 μm). **(I)** A graph showing percentage of 4-HNE-positive area to total area. Data are presented as mean ± SD and were analyzed by two-way ANOVA. *p < 0.05, **p < 0.01, ***p < 0.001 *versus* the Cont group in the PBS group. #*p* < 0.05, ##*p* < 0.01, ###*p* < 0.001 *versus* the Cont group in the APAP group.

Male offspring mice in prenatal MSCS groups exhibited increased hepatocellular necrosis and elevated serum ALT levels, particularly in the CS600 group, compared to APAP injected Cont group (Figures 2A,B). In contrast, we did not observe any significant differences in PBS injection groups. Such effects were also observed through TUNEL staining and subsequent analysis of its-positive cells (Figures 2C,D). Furthermore, mice without injury showed trends toward increased hepatic inflammation after prenatal MSCS exposure, which was consistent with necropsy examination of 2 weeks old offspring (Figure 1A). These patterns were evident in injured livers as determined by measurements of *Tnfα* and *Il1β* mRNA levels (Figure 2E).

It has been reported that infiltration of neutrophils is one of the key features of APAP-induced liver injury. They can be considered as a potential target for treating of APAP-induced ALI (Guo et al., 2021). Thus, we next evaluated hepatic accumulation of neutrophils using IHC staining targeting MPO, a well-known marker of neutrophil. As shown in Figures 2F,G, neutrophil infiltration was markedly increased following APAP injection. This effect was further enhanced in a dose-dependent manner by prenatal exposure to MSCS. Similar patterns were observed in IHC staining targeting 4-HNE, an indicator of oxidative stress (Figures 2H,I). Collectively, these results indicate that maternal smoking can accelerate the severity of APAP-induced hepatotoxicity by increasing oxidative stress and causing extensive hepatocellular necrosis. This in turn triggers an inflammatory response involving neutrophil recruitment in livers of mice exposed to prenatal MSCS.

### Maternal MSCS exposure regulates hepatic expression of APAP-metabolizing CYP enzymes in male offspring mice

**FIGURE 3:**
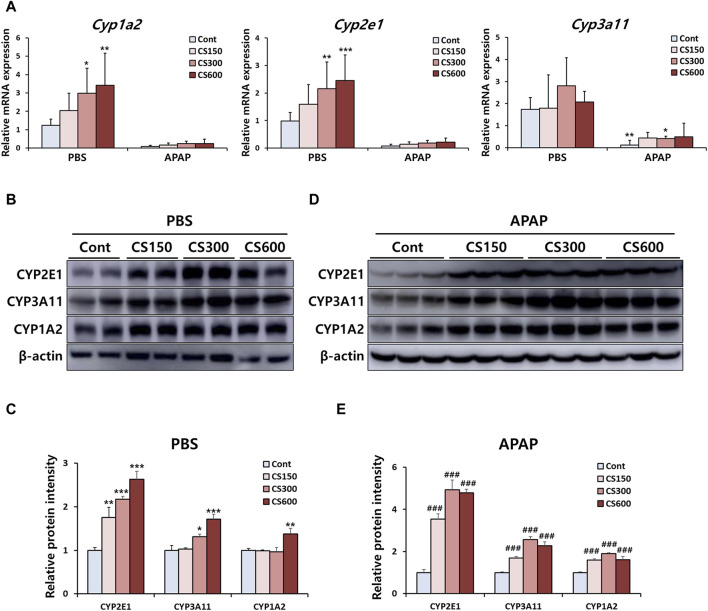
Maternal MSCS exposure regulates hepatic expression of APAP-metabolizing CYP enzymes in male offspring mice. **(A)** Hepatic mRNA levels of CYP enzymes determined by qRT-PCR. **(B–E)** Hepatic protein expression levels of CYP enzymes in PBS or APAP group determined by Western blotting with relative protein intensity quantified. Data are presented as mean ± SD and were analyzed by one-way (Panel C, E) or two-way ANOVA (Panel A). *p < 0.05, **p < 0.01, ***p < 0.001 *versus* the Cont group in the PBS group. ###*p* < 0.001 *versus* the Cont group in the APAP group.

The liver is the primary organ where CYP enzymes crucial for drug metabolism are predominantly found. CYP1A2, 2E1, and 3A11 are closely associated with the metabolism of APAP. An increase in their expression can raise the risk of APAP-induced liver injury (Bao et al., 2020). Consistent with previous findings (Figures 1C,D), mRNA expression levels of these CYPs were upregulated in livers of mice without injury due to prenatal exposure to MSCS (Figure 3A). We also observed similar patterns of increased protein levels for these enzymes, which were confirmed through Western blot analysis (Figures 3B,C). Although mRNA expression levels of these enzymes in livers of offspring exposed to MSCS *in utero* showed a trend towards increases, although this trend was not statistically significant. However, protein levels in livers were significantly increased due to maternal smoking (Figures 3D,E). These findings suggested that male offspring from mothers who smoked during pregnancy displayed altered hepatic expression levels of CYP enzymes, which could subsequently influence the severity of drug-induced liver injury in the future.

### Maternal MSCS exposure downregulates expression of miR-34a-5p in livers of offspring mice

**FIGURE 4:**
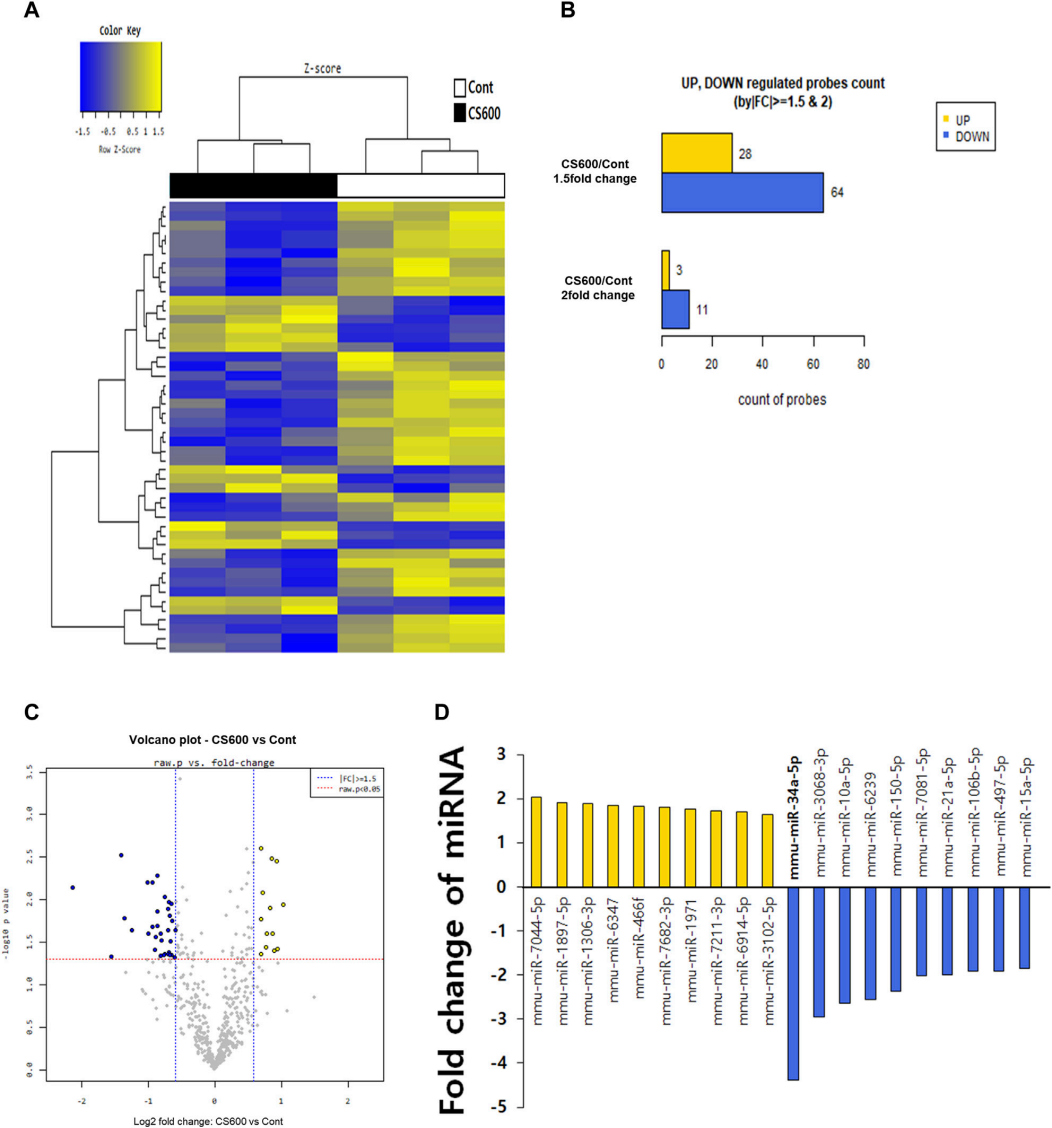
Maternal MSCS exposure downregulates expression of miR-34a-5p in livers of offspring mice. To determine which miRNAs were influenced by maternal MSCS exposure, a microarray was performed for livers of male offspring mice. **(A)** Cluster analysis classifying samples into groups based on miRNA expression levels in each sample. The dendrogram displays significantly different miRNA expression levels among samples. Yellow indicates high miRNA expression and blue shows relatively low miRNA expression. Only data with Z-score absolute values within 1.5 are shown. **(B)** Numbers of miRNAs that changed 1.5- or 2-fold in the CS600 group compared to the Cont group. **(C)** Volcano plots displaying differential expression based on fold-change and significance between two groups using a scatter plot view. **(D)** Top 10 differentially expressed miRNAs in the liver from the CS600 group compared to the Cont group.

A previous report has shown that whole-body exposure to environmental CS immediately after birth and for 2 weeks after weaning affects miRNA expression in livers and lungs of neonatal mice (Izzotti et al., 2010). This finding prompted us to investigate whether prenatal exposure to MSCS could also regulate hepatic miRNA expression in offspring mice. Hence, we carried out a microarray analysis in Cont and CS600 groups without any liver injuries to explore potential hepatic miRNAs that might be affected by maternal smoking. According to analysis results as shown in Figure 4A, we observed that among miRNAs with >1.5-fold change value, 28 miRNAs were upregulated and 64 miRNAs were downregulated. Furthermore, 3 miRNAs showed an increase of more than two-fold and 11 miRNAs exhibited a decrease of more than two-fold (Figure 4B). Based on these findings, we identified the ten most highly significantly upregulated or downregulated miRNAs in the CS600 group compared to the Cont group (Figures 4C,D). Among these miRNAs, the most profoundly altered one was miR-34a-5p with a fold change of −4.379 (*p* = 0.007). Therefore, miR-34a-5p was chosen for further research. In conclusion, prenatal exposure to MSCS can modulate hepatic miRNAs, particularly miR-34a-5p, in the offspring, potentially influencing future susceptibility to APAP-induced hepatotoxicity.

### Overexpression of miR-34a-5p attenuates harmful effects of maternal smoking on APAP-induced ALI in offspring mice

**FIGURE 5:**
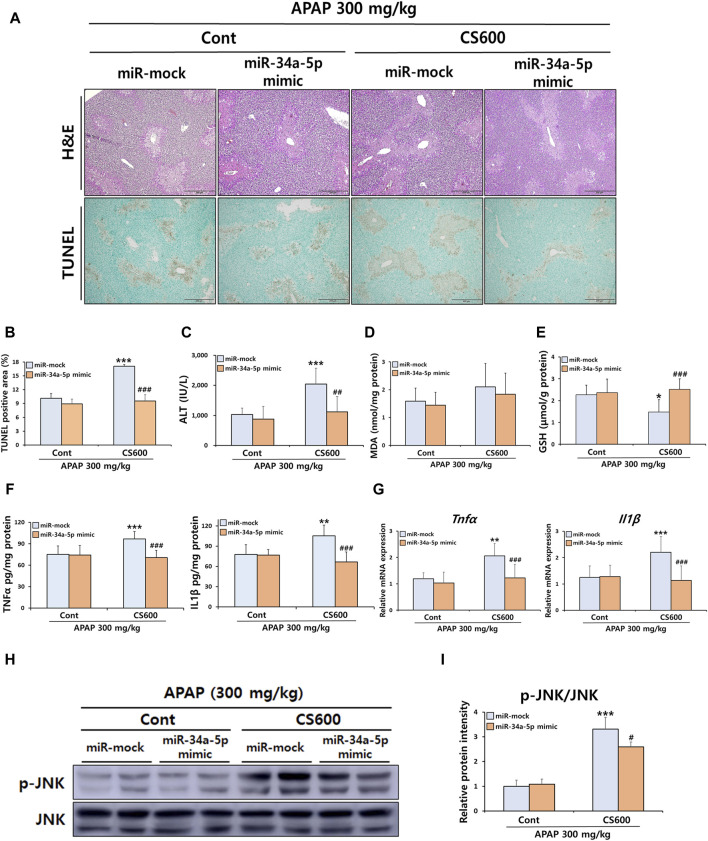
Overexpression of miR-34a-5p attenuates harmful effects of maternal smoking on APAP-induced ALI in offspring mice. Pregnant mice were exposed to MSCS (0 or 600 μg/L) for 2 h a day, 5 days a week for 2 weeks from day 6 to day 17 of gestation using a nose-only exposure system. At 4 weeks old, male offspring mice received a single i v. injection of either the miR-34a-5p mimic (1 nmol/mouse) or miR-mock. Mice were then given a single i.p. injection of APAP (300 mg/kg) at 24 h after the mimic injection. **(A)** Representative histological liver sections stained with H&E and TUNEL (Scale bar: 200 μm). **(B)** TUNEL-positive area graph. **(C)** Serum ALT levels. **(D,E)** Levels of MDA and GSH in hepatic proteins. Hepatic mRNA and protein expressions of pro-inflammatory cytokines were determined by ELISA **(F)** and qRT-PCR **(G)**. **(H,I)** Hepatic protein expression levels of p-JNK and JNK determined by Western blotting with relative protein intensity quantified. Data are presented as mean ± SD and were analyzed by two-way ANOVA. *p < 0.05, **p < 0.01, ***p < 0.001 *versus* the miR-mock group of the Cont group. #*p* < 0.05, ##*p* < 0.01, ###*p* < 0.001 *versus* the miR-mock group of the CS600 group.

We next evaluated the influence of reduced hepatic miR-34a-5p expression resulting from prenatal exposure to MSCS on APAP-induced hepatotoxicity in the offspring. To overexpress miR-34a-5p within the liver, we employed either a miR-34a-5p mimic or a miR-mock. To ascertain the efficiency of overexpression, mice were subjected to i.v. injection of either the miR-34a-5p mimic or the miR-mock at doses ranging from 0.5 to 10 nmol per mouse. Subsequently, we measured hepatic miR-34a-5p expression levels. Results revealed that treatment with the miR-34a-5p mimic at a dose of 1 nmol led to an approximately 4-fold increase in hepatic miR-34a-5p expression (Supplementary Figure S2A). Since miR-34a-5p was downregulated approximately 4-fold in livers of offspring by maternal smoking (Figure 4D), we conducted additional experiments using 1 nmol miR-34a-5p mimic, expecting that treatment with this mimic could restore decreased hepatic expression of miR-34a-5p in offspring caused by prenatal exposure to MSCS. We also hypothesized that overexpression of miR-34a-5p might abrogate adverse effects of maternal smoking on APAP-induced ALI in offspring mice. As expected, treatment of mice with 1 nmol of the miR-34a-5p mimic restored miR-34a-5p levels to normal levels, which had been reduced due to prenatal exposure to MSCS (Supplementary Figure S2B). Interestingly, overexpression of miR-34a-5p completely abolished detrimental impacts of *in utero* MSCS exposure on APAP-induced liver damage as evidenced by diminished levels of serum ALT and reduced hepatocellular necrosis (Figures 5A–C). While hepatic MDA levels were comparable across groups (Figure 5D), overexpression of miR-34a-5p restored hepatic levels of GSH, which were otherwise diminished in injured livers of offspring mice due to maternal smoking during pregnancy (Figure 5E). Furthermore, mRNA and protein levels of pro-inflammatory cytokines, including TNFα and IL1β, were dramatically decreased by overexpression of miR-34a-5p (Figures 5F,G). We then examined protein activation of JNK known to regulate inflammatory responses. Consistently, overexpression of miR-34a-5p regulated the severity of apoptosis by significantly reducing JNK phosphorylation in livers of offspring mice exposed to maternal MSCS during pregnancy (Figures 5H,I). Overall, overexpression of miR-34a-5p abolished detrimental effects of prenatal exposure to MSCS on APAP-induced ALI.

### miR-34a-5p regulates APAP-induced liver injury by affecting HNF4α signaling in mice

**FIGURE 6:**
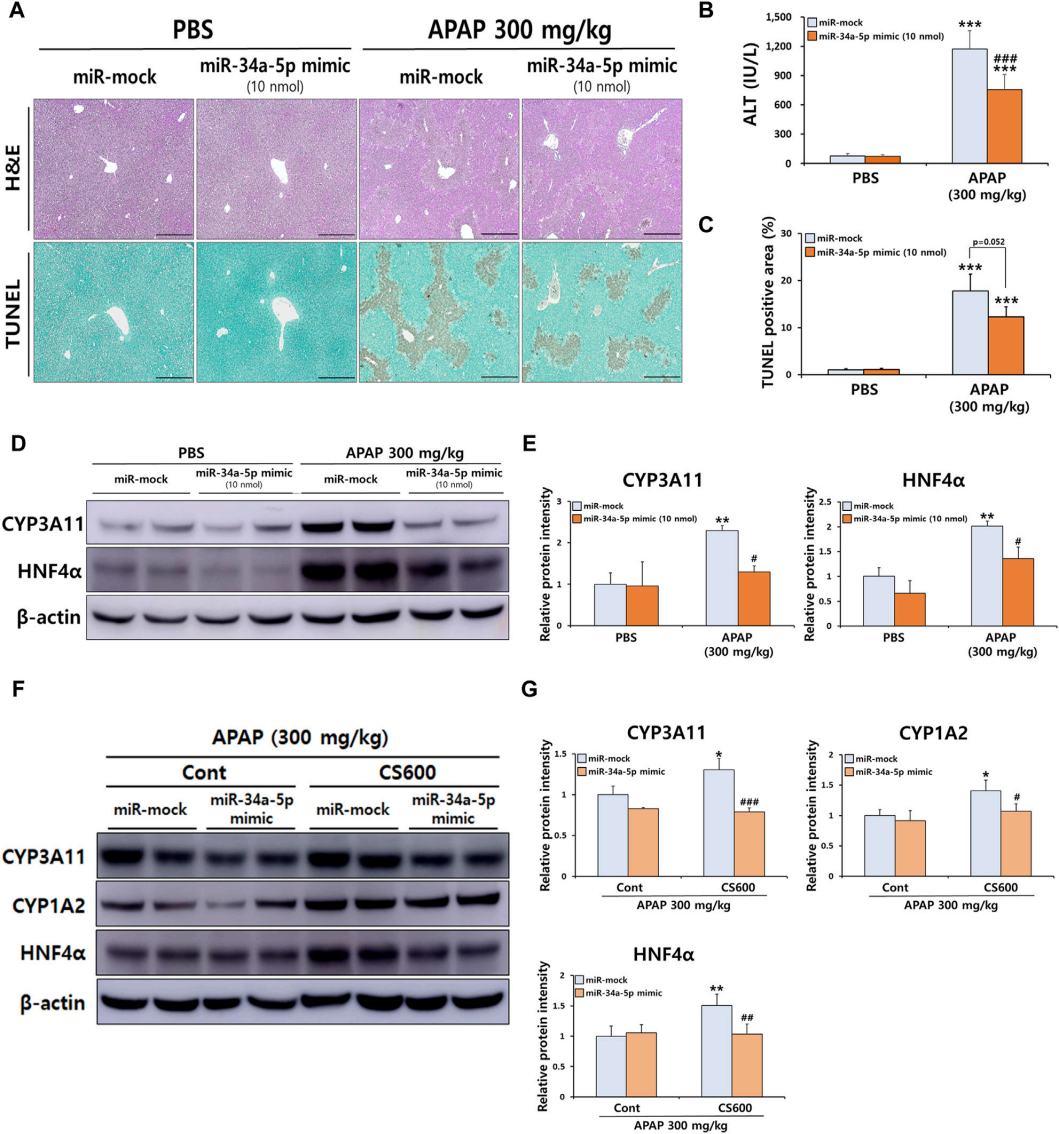
miR-34a-5p regulates APAP-induced liver injury by affecting HNF4α signaling in mice. Male mice at 4 weeks old received a single i.v. injection of either the miR-34a-5p mimic (10 nmol/mouse) or the miR-mock. Mice were then administered a single i.p. injection of APAP (300 mg/kg) at 24 h post-mimic injection. **(A)** Representative histological liver sections stained with H&E and TUNEL (Scale bar: 200 μm). **(B)** Serum ALT levels. **(C)** Graph showing TUNEL-positive area. **(D)** Hepatic protein expression levels of CYP3A11 and HNF4α were confirmed by Western blotting, and **(E)** relative protein intensities were quantified. **(F)** Hepatic protein expression levels of CYP3A11, 1A2, and HNF4α were confirmed by Western blotting, and **(G)** relative protein intensities were quantified in livers of offspring mice with prenatal MSCS exposure and subsequent APAP injection. Data are presented as mean ± SD and were analyzed by two-way ANOVA. **p < 0.01, ***p < 0.001 *versus* Cont group of PBS group. #*p* < 0.05, ###*p* < 0.001 *versus* Cont group of APAP group in Panel B,C,E. *p < 0.05, **p < 0.01 *versus* miR-mock group of Cont group. #*p* < 0.05, ##*p* < 0.01, ###*p* < 0.001 *versus* miR-mock group of CS600 group in Panel G.

Although overexpression of miR-34a-5p counteracted adverse effects of maternal smoking during pregnancy in mice with ALI, it failed to alleviate the severity of APAP-induced ALI (Figure 5). This result was inconsistent with a previous finding showing that miR-34a-5p exerted a protective role in liver injury (Win et al., 2019). Therefore, we next conducted additional experiments to determine whether miR-34a-5p could exert a protective role in APAP-induced ALI using a high dose of the miR-34a-5p mimic (10 nmol per mouse). Interestingly, the severity of APAP-induced hepatotoxicity was markedly decreased by overexpressing the miR-34a-5p mimic as confirmed by histopathological examination, TUNEL staining, and serum ALT measurement (Figures 6A–C). Our previous results indicated that maternal exposure to MSCS during pregnancy affected the expression of CYP1A2, 2E1, and 3A11 (Figures 1, 3) as well as the expression of miRNAs, particularly miR-34a-5p (Figure 4), in livers of offspring mice. To establish a connection between these findings, we then determined whether miR-34a-5p could influence the expression of these CYP enzymes in livers. As shown in Figures 6D,E, protein levels of CYP3A11 increased by APAP treatment were significantly reduced upon injection of the miR-34a-5p mimic. Furthermore, we assessed protein expression levels of HNF4α, a recognized target of miR-34a-5p known to play a role in regulating CYP enzymes (Zheng et al., 2022). Consistent with CYP3A11 findings, hepatic abundance of HNF4α also displayed a significant decrease upon overexpression of miR-34a-5p (Figures 6D,E). These findings were further supported by results presented in Figures 6F,G, providing evidence that protein levels of HNF4α targeted by miR-34a-5p and found to be elevated in injured livers of offspring mice due to prenatal MSCS exposure were notably diminished upon injection of the miR-34a-5p mimic. Similar trends were observed for CYP3A11 and 1A2 known to be downstream effectors of HNF4α. However, they showed no significant changes in Cont groups (Figures 6F,G).

### FA-mediated downregulation of miR-34a-5p sensitizes APAP-induced hepatotoxicity *in vitro*

**FIGURE 7:**
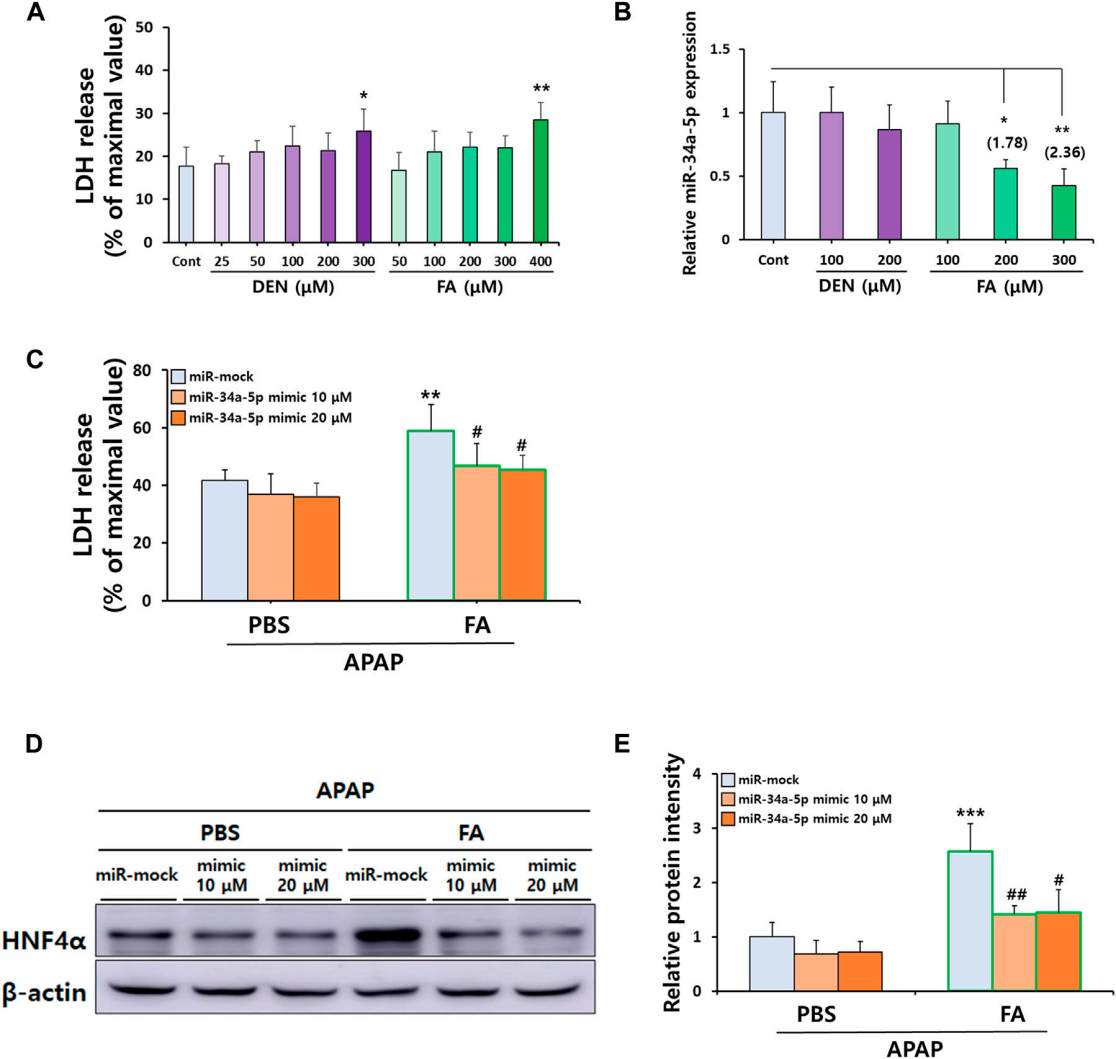
FA-mediated downregulation of miR-34a-5p sensitizes APAP-induced hepatotoxicity *in vitro*. **(A)** Non-toxic concentrations of DEN and FA in primary hepatocytes determined by LDH cytotoxicity assay. **(B)** Hepatic miR-34a-5p expression levels after DEN and FA treatment determined by qRT-PCR using TaqMan™ MicroRNA Assay Kit. U6 snRNA was used as an internal control for relative quantification of miRNA expression levels. **(C)** Effects of miR-34a-5p (10 or 20 μM) overexpression in primary hepatocytes isolated from mice on hepatotoxicity induced by APAP (5 mM) and FA (300 μM) treatment determined by LDH cytotoxicity assay. **(D,E)** Hepatic protein expression of HNF4α determined by Western blotting with relative protein intensity quantified. Data are presented as mean ± SD and were analyzed by one-way (Panel A,B) or two-way ANOVA (Panel C,E). *p < 0.05, **p < 0.01 *versus* Cont group in Panel A,B. **p < 0.01, ***p < 0.001 *versus* miR-mock group of APAP-PBS group and #*p* < 0.05, ##*p* < 0.01 *versus* miR-mock group of APAP-FA group in Panel C,E.

To replicate our *in vivo* observations, we investigated whether treatment with CS ingredients such as DEN and FA (Smith et al., 2000), affected the severity of APAP-induced hepatocellular death by modulating miR-34a-5p expression *in vitro* using primary hepatocytes. After treating cells with varying concentrations of DEN and FA, we found that the maximal non-toxic doses for DEN and FA were 200 μM and 300 μM, respectively (Figure 7A). Interestingly, only FA treatment at concentrations above 200 μM significantly reduced miR-34a-5p expression (Figure 7B). Moreover, treatment with miR-34a-5p mimics significantly counteracted deleterious effects of FA on APAP-induced hepatotoxicity (Figure 7C), effectively elevating miR-34a-5p expression without inducing cytotoxicity (Supplementary Figure S3A,B). Similar to *in vivo* results, protein expression levels of HNF4α increased by treatment with FA were significantly decreased by treatment with miR-34a-5p mimic, although no dose-dependent effect was observed (Figures 7D,E). Collectively, these data suggest that upregulation of miR-34a-5p can alleviate APAP-induced liver injury by modulating the HNF4α-CYP signaling axis in male offspring mice with maternal MSCS exposure.

## Discussion

Numerous studies have extensively investigated effects of maternal smoking during pregnancy on long-term health issues, particularly respiratory diseases. Several clinical research studies have found a correlation between maternal smoking and detrimental impacts on lung development, with these impacts subsequently increasing susceptibility to various respiratory disorders such as infections and asthma (Carroll et al., 2007; Milner et al., 2007; Zacharasiewicz, 2016). Consistently, it has been found that maternal smoking during pregnancy in mice can amplify oxidative stress and production of inflammatory cytokines in lungs of the offspring (Sukjamnong et al., 2017). Additionally, perinatal exposure to CS can exacerbate allergic reactions induced by house dust mites and asthma via immune modulation (Janbazacyabar et al., 2021). Moreover, fetal exposure to second-hand CS can suppress mucus production and augments Th2 polarization, resulting in increased susceptibility to allergic asthma and childhood respiratory infections in mice (Singh et al., 2011). Maternal smoking during pregnancy has detrimental effects on offspring mice, impacting not only their lungs, but also other organs. It has been documented that maternal smoking can induce alterations in proteome profile and cause mitochondrial defects in kidneys and livers of the offspring (Jagadapillai et al., 2012; Stangenberg et al., 2015; Neal et al., 2016; Li et al., 2019b). Moreover, maternal nicotine exposure can cause congenital heart defects and coronary artery malformation in fetal mice (Greco et al., 2022). Recent studies have emphasized that harmful effects of CS on the fetus during pregnancy are not limited to traditional cigarettes because e-cigarettes also pose risks. It has been shown that maternal exposure to electronic cigarette vapor has particularly evident adverse effects on kidneys and lungs of fetuses (Li et al., 2019a; Noël et al., 2020). Epigenetic alterations, including DNA methylation, are among reasons why maternal exposure to CS or e-cigarettes has harmful effects on offspring mice (Chen et al., 2018; Nguyen et al., 2018; Stangenberg et al., 2019). In line with these findings, our study further revealed that smoking during pregnancy could impact livers of offspring mice, especially in terms of miRNA expression and associated CYP enzyme levels. Consistently, prenatal CS exposure increased *Cyp2a5* gene expression and led to persistent and higher promoter methylation in neonates. This could result in heightened nicotine metabolism, making mice more susceptible to nicotine dependence later in life (Lkhagvadorj et al., 2020).

Interestingly, our results also revealed that maternal MSCS exposure made offspring mice more vulnerable to APAP-induced hepatotoxicity by regulating miRNA expression, especially miR-34a-5p. The role of miR-34a-5p has been investigated in various models of acute and chronic liver injury. Considering various results, the function of miR-34a-5p might vary depending on disease models and their progression stages. In non-alcoholic fatty liver disease with iron overload, silencing of miR-34a can increase Sirtuin 1 expression and consequently alleviate TG accumulation (Wang L. et al., 2020). Moreover, miR-34a has been reported to function as a pro-fibrosis factor that can promote alcohol-induced liver fibrosis by reducing senescence of hepatic stellate cells (HSCs) and conversely increasing hepatocyte senescence (Wan et al., 2017). However, some studies have shown evidence supporting hepatoprotective effects of miR-34a. Overexpression of miR-34a-5p has been found to be able to mitigate liver fibrosis development and progression by targeting Smad 4 in HSCs (Feili et al., 2018). Recent studies have reported that miR-34a-5p can protect the liver from Ischemia/Reperfusion injury through downregulating HNF4α (Zheng et al., 2022). Furthermore, markedly enhanced liver injuries in mice with hepatocytes-specific miR-34a-5p deficiency in the APAP-induced ALI model strongly suggest that miR-34a-5p might play a critical role in the improvement of ALI (Win et al., 2019). This hepatoprotective function of miR-34a is consistent with our results showing that an increase of APAP-induced ALI by *in utero* CS exposure was alleviated by miR-34a-5p overexpression.

Mechanistically, we found that overexpression of miR-34a-5p significantly reduced protein levels of HNF4α and CYP3A11, leading to a decrease in the severity of APAP-induced ALI. Our results were further supported by recent studies indicating that HNF4α is a target of miR-34a-5p (Xu et al., 2015; Li and Chen, 2019). Moreover, CYP enzymes including CYP1A2 and CYP3A11 known to play a pivotal role in drug metabolism are regulated by HNF4α (Xu et al., 2022). Additionally, overexpression of HNF4α has been found to exacerbate APAP-induced hepatotoxicity (An et al., 2019). Hence, the miR-34a-5p/HNF4α/CYPs axis might be crucial for drug metabolism and associated liver injuries. Consistent with this notion, our data showed that maternal smoking decreased expression levels of miR-34a-5p in offspring mice. This led to increased expression levels of HNF4α and CYP enzymes in the liver, making the offspring more susceptible to APAP overdose. Conversely, overexpressing miR-34a-5p completely mitigated adverse effects of prenatal exposure to CS on offspring mice owing to reduced levels of HNF4α, CYP1A2, and CYP3A11.

Based on our findings that maternal MSCS exposure could alter miRNA expression and affects subsequent ALI in offspring, it is plausible that many CS components exposed to pregnant mice are transmitted to the offspring, leading to a variety of unwanted side effects. Considering this, we endeavored to identify components that might downregulate the expression of miR-34a-5p in the liver of the offspring. Previous study has reported that various chemicals such as nicotine, acetaldehyde, isoprene, and benzene are present in MSCS generated by the ISO standard 3,308 method (Jaccard et al., 2019). Among many components of CS, we selected DEN and FA, both known to cross the placenta in mice (Bolognesi et al., 1988; Katakura et al., 1993; Monfared, 2014). DEN is known for its hepatotoxic effects and its potential to induce liver cancer. It undergoes metabolism by the enzyme CYP2E1, which is implicated in ROS production (Tolba et al., 2015). FA is recognized as a more potent carcinogen than DEN. It is present in the diet and ubiquitously present in the environment as well as in the CS. It is also found throughout the human body, participating in cellular metabolic processes such as histone and DNA demethylation reactions as well as the one-carbon cycle (Umansky et al., 2022). Prior research studies have indicated that FA exposure can modify miRNA expression across a range of cells and tissues (Rager et al., 2011; Rager et al., 2014). In line with this, our findings intriguingly revealed that FA treatment, but not DEN treatment, significantly diminished miR-34a-5p expression in primary hepatocytes. Moreover, while FA treatment enhanced APAP-induced hepatotoxicity, this detrimental effect was counteracted by overexpression of miR-34a-5p. Although FA treatment could not fully mimic effects of maternal smoking and MSCS, our findings indicate that FA can exacerbate APAP-induced hepatotoxicity *in vitro*, primarily through modulation of miR-34a-5p expression, consistent with *in vivo* observations. These findings are further supported by changes in protein expression of HNF4α, a downstream target of miR-34a-5p.

It has been already known that males tend to be less affected by hormones than females (Verthelyi, 2001). There is also a gender difference in basal expression of CYPs involved in APAP metabolism. For example, CYP1A2 and 2E1 are more active in males, whereas CYP3A4 displays an elevated activity in females (Scandlyn et al., 2008). Consistently, gender differences exist in susceptibility to ALI following APAP overdose, with male mice exhibiting more severe APAP-induced ALI than female mice (Du et al., 2014). Furthermore, one study has reported that maternal smoking has a greater impact on male fetuses than on female fetuses (Zaren et al., 2000). This finding is further supported by a recent study showing that maternal smoking has negative effects on lung health of fetuses, with female fetuses seeming to be less affected by adverse effects of maternal smoking than male fetuses (Wang B. et al., 2020). Although we focused on male offspring mice exposed to maternal MSCS in this study, future research should explore potential sex differences in effects of maternal smoking on the onset of ALI in offspring mice.

In conclusion, this is the first study to offer direct evidence that smoking during pregnancy can exacerbate drug-induced liver injury in offspring mice later in life by modulating hepatic miRNAs. Our findings strongly underscore the importance of caution concerning the transmission of toxicants from mother to fetus. We hope that our findings will enhance support for smoking cessation in pregnant smokers.

## Data Availability

The data presented in the study are deposited in the NCBI’s Gene Expression Omnibus 435 (GEO) repository, accession number GSE272826.
