# Supplementary material for: Prenatal cigarette smoke exposure sensitizes acetaminophen-induced liver injury by modulating miR-34a-5p in male offspring mice
Source: Front Cell Dev Biol. 2024 Jul 30;12:1393618. doi: 10.3389/fcell.2024.1393618 (PMC11319911; doi:10.3389/fcell.2024.1393618)
Supplement: Supplementary file 2 [file DataSheet1.docx]

Supplementary Figures

**
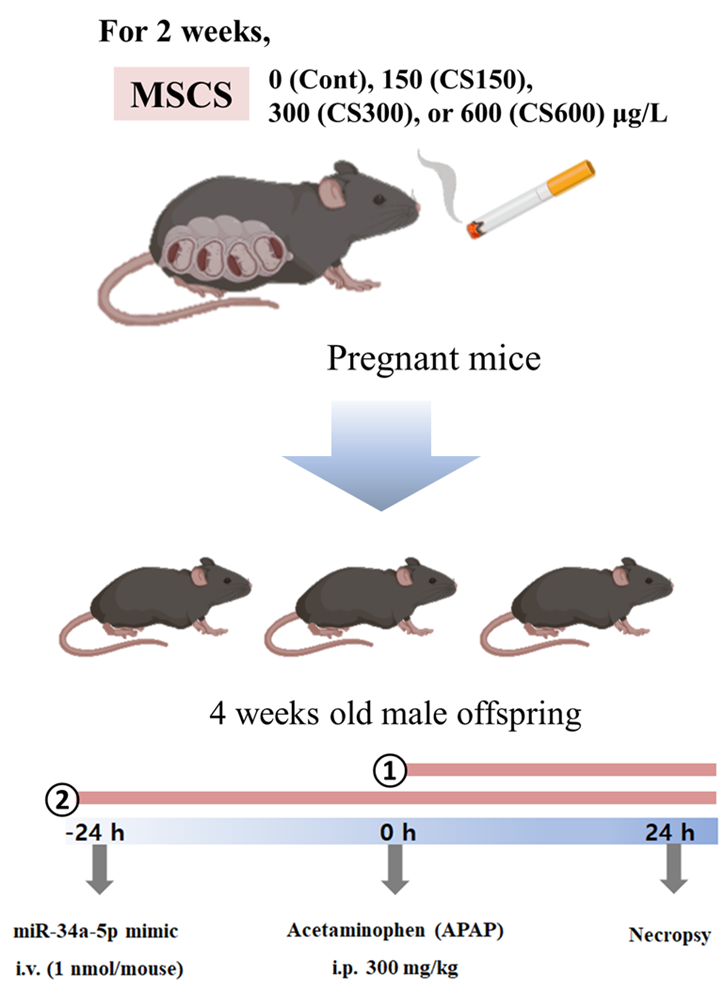
**

**Supplementary Figure S1.** Overall procedure of experiment.

In the first experiment, pregnant mice were exposed to an indicated concentration of MSCS from day 6 to day 17 of gestation using a nose-only exposure system. Male offspring mice were injected with APAP to induce ALI. In the second study, pregnant mice were exposed to MSCS at 0 or 600 μg/L using the same method. These male offspring mice received either the miR-mock or miR-34a-5p mimic at 24 hours before APAP treatment. Samples were then collected at 24 hours after APAP injection.

| **A** | **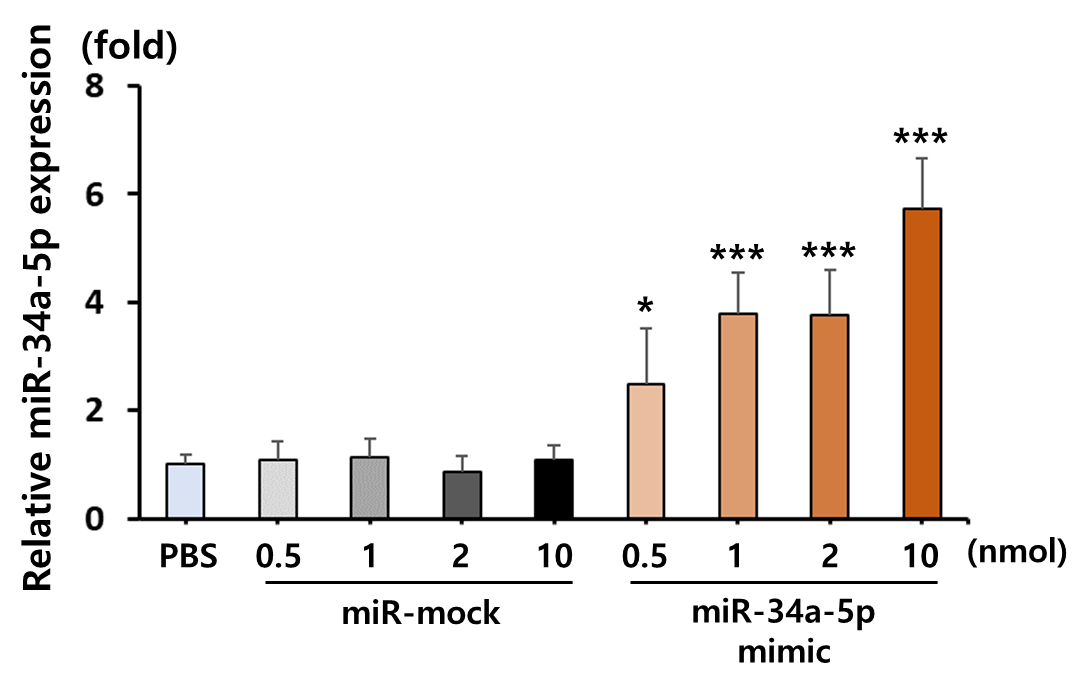** |
| --- | --- |
| **B** | **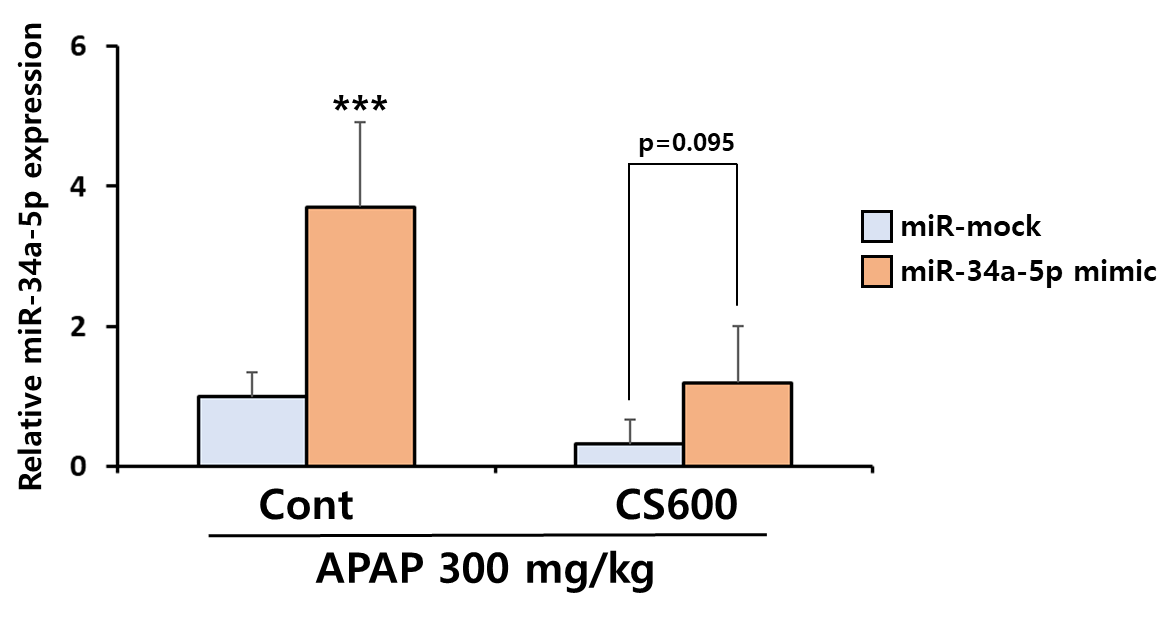** |

**Supplementary Figure S2.** Relative mRNA expression of miR-34a-5p.

(A) Mice were injected with PBS, miR-mock, or different doses of miR-34a-5p mimic to assess hepatic expression levels of miR-34a-5p. Data are presented as mean ± SD and were analyzed by one-way ANOVA. * *p* < 0.05, *** *p* < 0.001 versus the PBS group. (B) After injection of APAP (300 mg/kg) and miR-34a-5p mimic (1 nmol) into offspring mice exposed to prenatal MSCS, levels of miR-34a-5p were determined. Data are presented as mean ± SD and were analyzed by two-way ANOVA. *** *p* < 0.001 versus miR-mock group of Cont group.

| **A** | **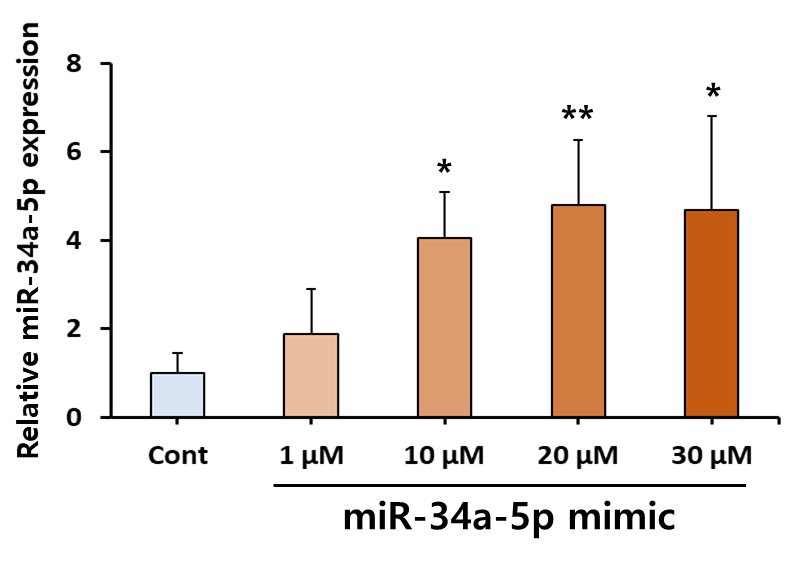** |
| --- | --- |
| **B** | **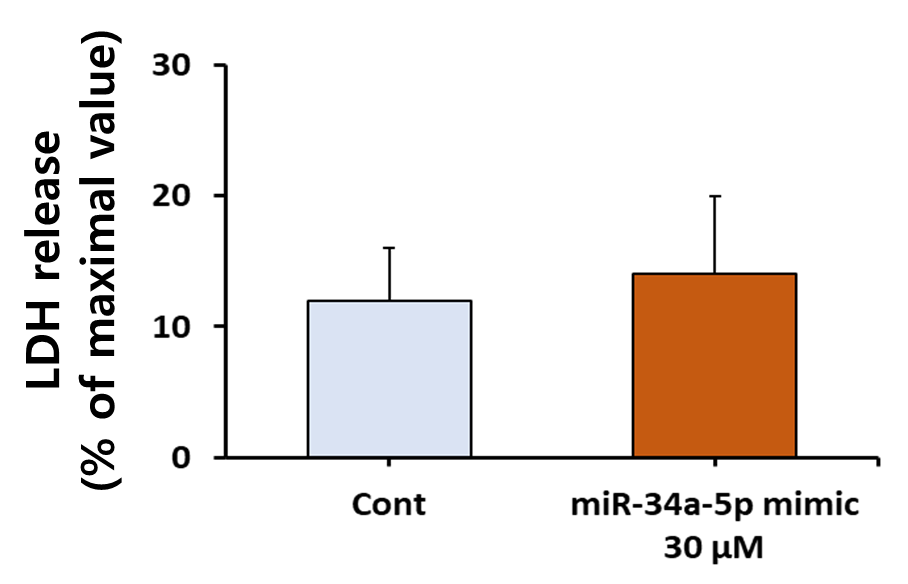** |

**Supplementary Figure S3.** Expression levels of miR-34a-5p in primary hepatocytes following treatment with miR-34a-5p mimic.

(A) Primary hepatocytes isolated from mice were treated with various concentrations of miR-34a-5p mimics to overexpress miR-34a-5p. Relative miR-34a-5p expression was significantly increased at doses above 10 μM. (B) The maximum dose of 30 μM mimic did not show cytotoxicity in the LDH assay. Data are presented as mean ± SD and were analyzed by one-way ANOVA. * *p* < 0.05, ** *p* < 0.01 versus the Cont group.
